# Supplementary material for: Spatial and temporal distribution characteristics and influencing factors of cultural heritage: A case of the Grand Canal (East Zhejiang section)-maritime silk road
Source: PLoS One. 2025 Feb 12;20(2):e0318529. doi: 10.1371/journal.pone.0318529 (PMC11819555; doi:10.1371/journal.pone.0318529)
Supplement: Attachment — Submitted filename: Response to Reviewers.docx [file pone.0318529.s003.docx]

Response to the Manuscript entitled “Spatial and temporal distribution characteristics and influencing factors of cultural heritage: a case of the Grand Canal (East Zhejiang section)-Maritime Silk Road”

Dear editor:

I would like to request you to consider the attached manuscript entitled “Spatial and temporal distribution characteristics and influencing factors of cultural heritage: a case of the Grand Canal (East Zhejiang section)-Maritime Silk Road” for publication in *PLOS ONE* as an original research article. This letter certifies that the content of this manuscript is original and is not submitted to or published in any other journal or publication. Each author represented has contributed significantly to the work and agrees to the submission of the manuscript.

The purpose of this study is to investigate the cultural heritage under the joint influence of the Grand Canal and the Maritime Silk Road. In this study, we focus on the evolutionary patterns of cultural heritage based on the synergistic development of the Grand Canal and the Maritime Silk Road from the historical and cultural perspectives, which is more distinctive than other articles. I believe that the findings of this study are relevant to the scope of your journal and will be of interest to its readership.

We sincerely appreciate the editor’s time and thoughtful consideration of our manuscript. Thank you for the invaluable insights and guidance provided.

**As requested by the journal, our response is as follows:**

1. Please ensure that your manuscript meets PLOS ONE's style requirements, including those for file naming. The PLOS ONE style templates can be found at

https://journals.plos.org/plosone/s/file?id=wjVg/PLOSOne_formatting_sample_main_body.pdf and

https://journals.plos.org/plosone/s/file?id=ba62/PLOSOne_formatting_sample_title_authors_affiliations.pdf

**Response**: We have revised the manuscript according to PLOS ONE style templates.

2. Thank you for stating the following financial disclosure:

“This work was funded by the Key Research and Development Program of Ningbo, Grant No. 2023Z137.”

Please state what role the funders took in the study.  If the funders had no role, please state: "The funders had no role in study design, data collection and analysis, decision to publish, or preparation of the manuscript."

If this statement is not correct you must amend it as needed.

Please include this amended Role of Funder statement in your cover letter; we will change the online submission form on your behalf.

**Response**: Thank you for the reminder, our funder was not involved in our research, so our funding statement is: “The funders had no role in study design, data collection and analysis, decision to publish, or preparation of the manuscript.”

3. Thank you for stating the following in the Acknowledgments Section of your manuscript:

“This work was funded by the Key Research and Development Program of Ningbo, Grant No. 2023Z137.”

We note that you have provided funding information that is currently declared in your Funding Statement. However, funding information should not appear in the Acknowledgments section or other areas of your manuscript. We will only publish funding information present in the Funding Statement section of the online submission form.

Please remove any funding-related text from the manuscript and let us know how you would like to update your Funding Statement. Currently, your Funding Statement reads as follows:

“This work was funded by the Key Research and Development Program of Ningbo, Grant No. 2023Z137.”

Please include your amended statements within your cover letter; we will change the online submission form on your behalf.

**Response**: Thank you for the reminder, we have removed the funding information from the Acknowledgements section and we do not need to update the funding statement.

4. We note that your Data Availability Statement is currently as follows: [All relevant data are within the manuscript and its Supporting Information files.]

Please confirm at this time whether or not your submission contains all raw data required to replicate the results of your study. Authors must share the “minimal data set” for their submission. PLOS defines the minimal data set to consist of the data required to replicate all study findings reported in the article, as well as related metadata and methods (https://journals.plos.org/plosone/s/data-availability#loc-minimal-data-set-definition).

For example, authors should submit the following data:

- The values behind the means, standard deviations and other measures reported;

- The values used to build graphs;

- The points extracted from images for analysis.

Authors do not need to submit their entire data set if only a portion of the data was used in the reported study.

If your submission does not contain these data, please either upload them as Supporting Information files or deposit them to a stable, public repository and provide us with the relevant URLs, DOIs, or accession numbers. For a list of recommended repositories, please see https://journals.plos.org/plosone/s/recommended-repositories.

**Response**: Thank you for the reminder and we will submit the cultural heritage dataset as supporting information.

5. We note that Figures 1, 2, 4, 5, 6, 7, 8 and 9 in your submission contain [map/satellite] images which may be copyrighted. All PLOS content is published under the Creative Commons Attribution License (CC BY 4.0), which means that the manuscript, images, and Supporting Information files will be freely available online, and any third party is permitted to access, download, copy, distribute, and use these materials in any way, even commercially, with proper attribution. For these reasons, we cannot publish previously copyrighted maps or satellite images created using proprietary data, such as Google software (Google Maps, Street View, and Earth). For more information, see our copyright guidelines: http://journals.plos.org/plosone/s/licenses-and-copyright.

We require you to either (1) present written permission from the copyright holder to publish these figures specifically under the CC BY 4.0 license, or (2) remove the figures from your submission:

1. You may seek permission from the original copyright holder of Figures 1, 2, 4, 5, 6, 7, 8 and 9 to publish the content specifically under the CC BY 4.0 license.

We recommend that you contact the original copyright holder with the Content Permission Form (http://journals.plos.org/plosone/s/file?id=7c09/content-permission-form.pdf) and the following text:

“I request permission for the open-access journal PLOS ONE to publish XXX under the Creative Commons Attribution License (CCAL) CC BY 4.0 (http://creativecommons.org/licenses/by/4.0/). Please be aware that this license allows unrestricted use and distribution, even commercially, by third parties. Please reply and provide explicit written permission to publish XXX under a CC BY license and complete the attached form.”

Please upload the completed Content Permission Form or other proof of granted permissions as an "Other" file with your submission.

In the figure caption of the copyrighted figure, please include the following text: “Reprinted from [ref] under a CC BY license, with permission from [name of publisher], original copyright [original copyright year].”

2. If you are unable to obtain permission from the original copyright holder to publish these figures under the CC BY 4.0 license or if the copyright holder’s requirements are incompatible with the CC BY 4.0 license, please either i) remove the figure or ii) supply a replacement figure that complies with the CC BY 4.0 license. Please check copyright information on all replacement figures and update the figure caption with source information. If applicable, please specify in the figure caption text when a figure is similar but not identical to the original image and is therefore for illustrative purposes only.

**Response**: Thank you for your consideration. The maps used in Figures 1, 2, 4, 5, 6, 7, and 8 in the manuscript, both in shape and dem images, are publicly used by the Chinese government, and can be downloaded and used by anyone without copyright dispute. Therefore, we do not need authorization to use them, and there will be no copyright disputes after publication. The data we use comes from the Ministry of Natural Resources of China, and is free to use by all citizens of the country at http://bzdt.ch.mnr.gov.cn/. There are also many papers that have now used these data. At the same time, we did not use the self-help mapping function of the website, but used ArcGIS software to draw the images on the basis of the base map without any modification, which is in full compliance with the requirements of the website or the requirements of the Ministry of Natural Resources of China. Moreover, we will make a similar statement in the title of the picture: the image is made based on the standard map review number GS (xxxx) xxxx downloaded from the website of Standard Map Service of the Ministry of Natural Resources of China, with no modification of the base map, and drawn by ArcGIS software. In addition, if these images are deleted, we will not be able to express our content correctly.

6. Please review your reference list to ensure that it is complete and correct. If you have cited papers that have been retracted, please include the rationale for doing so in the manuscript text, or remove these references and replace them with relevant current references. Any changes to the reference list should be mentioned in the rebuttal letter that accompanies your revised manuscript. If you need to cite a retracted article, indicate the article’s retracted status in the References list and also include a citation and full reference for the retraction notice.

**Response**: Thank you for the reminder, we have checked the reference list and it does not cite the retracted paper. At the same time, there are no additions or subtractions to the references cited, except for a relatively minor change in the format of the reference markers.

**Review Comments to the Author**

Please use the space provided to explain your answers to the questions above. You may also include additional comments for the author, including concerns about dual publication, research ethics, or publication ethics. (Please upload your review as an attachment if it exceeds 20,000 characters)

Reviewer #1: This paper focuses on the cultural heritage under the joint influence of the Grand Canal (East Zhejiang section) and the Maritime Silk Road, and explores its spatial and temporal evolution patterns and related influencing factors. This work shows a certain logic, substantive coherence, and has important practical significance. I will recommend it to be published at Plos ONE. However, the manuscript still has little issues that should be addressed through appropriate modifications. Thus, minor revision is needed.

1. L128: Please specify which part of the river system data was used.

**Response**: Thanks for the heads up, this omission has been added.

The Digital Elevation Model (DEM) data and the river data were obtained from the Geospatial Data Cloud (http://www.gscloud.cn/) and the Open Street Map (https://www.openstreetmap.org/), respectively. In addition, for the river data, irrelevant factors such as drains and sewers were eliminated and naturally occurring rivers were selected.

2. L134: Please check the format of the formulas for each method in Research method and some of the formulas in its notes.

**Response**: Thanks for the heads up. The formatting of the equation section in the research method has all been rechecked. Both the numbered equations and inline equations have been edited by applying the Mathtype, which is consistent with the formatting sample provided by *Plos ONE*. Therefore, there is no need to revise the section.

3. L235: The figure uses symbols of different shapes to mark out the different regions, which is not intuitive enough. It is recommended to mark them with boxes of different colors.

**Response**: Thanks to your suggestion, we have redrawn Figure 4.

**Fig 4. Overall kernel density of the Grand Canal (East Zhejiang section)-Maritime Silk Road cultural heritage.** (The blue box indicates Three-River Estuary, the green box indicates Yuyao city center, and the red box indicates Xiangshan Shipu fishing port.)

4. L235: Please keep the kernel density values in the figure to two decimals only and do the same for the subsequent kernel density figures.

**Response**: Thank you for your suggestions. Because some densities are so small that retaining two decimals is not appropriate, we have retained two significant figures for all density values.

**Fig 4. Overall kernel density of the Grand Canal (East Zhejiang section)-Maritime Silk Road cultural heritage.** (The blue box indicates Three-River Estuary, the green box indicates Yuyao city center, and the red box indicates Xiangshan Shipu fishing port.)

**Fig 5. Kernel density of different types of the Grand Canal (East Zhejiang section)-Maritime Silk Road cultural heritage.**

**Fig 6. Kernel density of the Grand Canal (East Zhejiang section)-Maritime Silk Road cultural heritage in different periods.**

5. L480: The third column of table 10 has too much to say about impacts, and it is suggested that cuts be made to streamline the sentences.

**Response**: This part has been redacted.

**Table 10. Regional cultural impacts and representative cultural heritages.**

| Cultures | Period of emergence | Impacts | Representative cultural heritages |
| --- | --- | --- | --- |
| Hemudu Culture | Neolithic period | Hemudu Culture marks the cultural origins of the Yangtze River Basin, shaping an oceanic space that fostered a diverse Chinese civilization and laid the foundation for the Maritime Silk Road. | Marine culture sites, such as Jingtoushan Site. |
| Ancient Yue Culture | Warring States period | Ancient Yue Culture, a continuation of Hemudu Culture, is a maritime culture with an outward and dispersive nature, profoundly influencing the Maritime Silk Road. | Urban sites and tomb complexes, such as Gouzhang Former City Site. |
| Buddhist Culture | Late Eastern Han Dynasty | Buddhist Culture originated in India and spread to northern China and East Asia via the Grand Canal and the Maritime Silk Road. | Temple-type ancient buildings, such as Tiantong Temple, Ashoka Temple, and Baoguo Temple. |
| Zhedong Academic Culture | Northern Song Dynasty | The Zhedong school, led by Wang Yangming and Huang Zongxi, promoted pragmatism and the importance of industry and commerce, sparking early democratic ideas. These ideas spread via the Grand Canal and Maritime Silk Road, profoundly influencing regional culture and Ningbo’s merchant prosperity. | Ancient buildings, such as Baiyun Village and Wang Yangming’s Former Residence. |
| Book Collection Culture | Northern Song Dynasty | Ningbo’s Book Collection Culture, shaped by private collections passed down through generations, reflects the city’s long-standing pursuit of knowledge. | Book-collecting pavilions, such as Tianyi Pavilion, Wanjuan Tower, and Wugui Hall. |
| Mazu Culture | Late Northern Song Dynasty | Mazu, a goddess of the sea and key figure in China’s maritime culture, is a central folk belief tied to the nation’s peaceful diplomacy, maritime transport, and trade for over a thousand years. | Tianhou palace buildings, such as Qing’an Guild Hall. |
| Coastal Defense Culture | Ming Dynasty | Ningbo’s Coastal Defense Culture, shaped by its resistance to British, French, and Japanese forces, is a vital part of Maritime Silk Road history. | Coastal defense sites, such as Zhenhaikou Coast Defense Site. |
| Merchant Gang Culture | Late Ming Dynasty | The Grand Canal and Maritime Silk Road fostered the rise of Ningbo Merchants, shaping their mercantilism, pioneering spirit, and global outlook. | Guild hall buildings, such as Qianye Guild Hall and Anlan Guild Hall. |

6. L628: Conclusions (3) and (4) have too many words and it is suggested to simplify them.

**Response**: Thanks for your suggestions, we have simplified conclusions (3) and (4), and additionally (2).

(2) The cultural heritage shows a pattern of large agglomeration and small dispersion, with the Three-River Estuary as the core high-density area. Different types of heritage display distinct distribution patterns. Ancient buildings, sites, tombs, and grotto temples and stone carvings show an aggregation-random trend, while modern important historical sites and representative buildings exhibit aggregation, and others are randomly distributed. Ancient buildings and modern important sites and representative buildings’ distribution mirrors the overall trend. Ancient sites cluster around the Yuyao Jiangbei junction, southern Haishu, and Dongqian Lake. Ancient tombs are more dispersed, with a core in northern Haishu. Grotto temples and stone carvings are concentrated in Haishu, the Three-River Estuary, and the Yinzhou Beilun junction. Overall, the heritage concentration is strongest near the Three-River Estuary, decreasing with distance.

(3) The distribution of cultural heritage varies by historical period. The closer the period, the more concentrated the heritage in the Three-River Estuary, particularly since the Ming to Qing periods. Pre-Qin heritage is mainly Neolithic settlements, while Qin-Tang heritage centers on Yue kiln sites. Song-Yuan heritage diversifies, mostly in north-central Ningbo. The Ming to Qing and modern periods are dominated by ancient buildings and important historical sites and representative buildings, all concentrated around the Three-River Estuary. The SDE azimuths range from 138° to 148.5°, indicating a northwest-southeast distribution of heritage. Over time, the center of gravity shifted from the south-southeast to west-north, converging toward the Three-River Estuary, with influence from the Grand Canal and Maritime Silk Road.

(4) The spatial-temporal distribution of the Grand Canal (East Zhejiang section)-Maritime Silk Road heritage is shaped by natural factors (topography, elevation, slope, slope aspect, rivers) and human factors (politics, population, culture). Heritage tends to cluster in low-elevation, gentle slope areas with strong hydrophilicity. However, the influence of slope aspect is negligible. While natural factors play a supporting role, human factors drive the evolution of heritage distribution.

Reviewer #2: This manuscript analyzes the spatial-temporal distribution characteristics and influencing factors of cultural heritage, using the Grand Canal (East Zhejiang section) in China as a case study. The findings have positive implications for the high-quality and sustainable development of the Grand Canal and the Maritime Silk Road. Specific suggestions are as follows:

(1) The description of the study area in the manuscript is unclear. For example, in the title, ‘a case of the Grand Canal (East Zhejiang section) - Maritime Silk Road’ is vague;

**Response**: We have modified it in the Research area section to make it seem clearer.

Located on the Ningshao Plain in the eastern part of Zhejiang Province (Fig. 1), Ningbo, as the southernmost city of the Grand Canal and a key starting port of the Maritime Silk Road, holds a unique historical position. At the opening ceremony of the Belt and Road Forum for International Cooperation, General Secretary Xi Jinping stated that the ancient ports in Ningbo and other places are “living fossils” that record the history of the ancient Silk Road. Ningbo has a long history and is the birthplace of the Hemudu Culture, which represents over 7,000 years of civilization [30]. Due to its unique historical background and geographic location, Ningbo has developed a series of cultures with regional characteristics, mainly the Grand Canal culture and the Maritime Silk Road culture. As a carrier of cultural dissemination and historical memory, the cultural heritage in Ningbo bears the imprints of different eras, making it quintessential area for researching the cultural heritage of the Grand Canal and the Maritime Silk Road. As a carrier of cultural transmission and historical memory, cultural heritage carries the imprints of different eras. Therefore, Ningbo is selected in this study as a case to explore the cultural heritage of the Grand Canal (East Zhejiang section)-Maritime Silk Road.

(2) The quality of all figures is too low. It is recommended to optimize and enhance the figures, add latitude and longitude grids, and include other necessary elements;

**Response**: Thank you for your suggestion, we have optimised and redrawn all the figures, while including the necessary elements. These figures will be uploaded separately in the submission system.

(3) Please add a description of the reliability of the underlying cultural heritage distribution data;

**Response**: Thanks for the heads up, we have added the description.

Data attributes of cultural heritage include name, era, type, latitude and longitude coordinates, address, protection level, and other relevant information of the cultural heritage. The geographic coordinates of cultural heritage sites were primarily collected using Global Positioning System (GPS) during field trips. Some hard-to-reach cultural heritage sites had their coordinates obtained by applying Baidu map coordinate picking. Additionally, Google Earth was utilized to cross-check these coordinates. Excel and ArcGIS10.8 software were used to establish the database of the cultural heritage of the Grand Canal (East Zhejiang section)-Maritime Silk Road and create the spatial distribution map of the cultural heritage (Fig. 2).

(4) It is suggested to refine the language and expressions throughout the manuscript, as many parts contain obscure language and errors.

**Response**: Thank you for your suggestions, we have rechecked and revised the writing style throughout.

In **Abstract**:

The Grand Canal and the Maritime Silk Road in China are globally significant cultural routes, which have contributed a wealth of cultural heritage through their historical development.

Focusing on the Ningbo area, this study analyzes the spatial and temporal distribution of 1,755 cultural heritage sites over five historical periods and explores the influencing factors through spatial and statistical analysis.

The results show that: (1) Ancient buildings, along with modern important historical sites and representative buildings, are the most numerous.

In **Introduction**:

The distribution of cultural heritage worldwide reflects the complex pathways of human social development, geographic changes, and historical evolution, showcasing diverse temporal and spatial characteristics.

This study will help clarify the development of these two cultural routes and foster cultural preservation.

In **Materials and methods**:

Located on the Ningshao Plain in the eastern part of Zhejiang Province (Fig. 1), Ningbo, as the southernmost city of the Grand Canal and a key starting port of the Maritime Silk Road, holds a unique historical position.

The geographic coordinates of cultural heritage sites were primarily collected using Global Positioning System (GPS) during field trips.

Some cultural heritage items are listed at both the national and provincial levels, and some single items include multiple geographically distant sites. To more accurately study the spatial distribution characteristics of the cultural heritage, this study treats duplicated items as a single item, and single items containing multiple cultural heritages as multiple items.

The SDE captures the central tendency, dispersion, and directional patterns of spatial elements, enabling the analysis of cultural heritage distribution over different periods [34].

In **Result**:

The elevation statistics of the cultural heritage sites reveal a distinct distribution pattern (Table 5).

In **Discussion**:

The Grand Canal and the Maritime Silk Road, both crucial trade and cultural exchange routes in ancient China and globally, share a close interrelationship.

The Grand Canal and the Maritime Silk Road are not only historically interconnected but have also maintained significant relevance in modern society.

Reviewer #3: The article analyzes the characteristics of spatial and temporal distribution of cultural heritage from the perspective of geography, which is innovative and the results of the study are of practical significance. The regulations are clear and the logic is rigorous, but the manuscript still has the following problems, and it is suggested that it be revised and re-reviewed:

1. it is suggested to modify the overall structure by dividing the first introductory part into two parts, namely, research background and literature review, and combining the second part and the third part into a complete results part.

**Response**: Thank you for your suggestions; we have adjusted the structure of the article. Because the structure of the article is not easy to show, please see the word document called ‘Revised Manuscript with Track Changes’ for details.

2. suggested deletions to the abstract

**Response**: Thank you for your suggestions, we have condensed the abstract.

The Grand Canal and the Maritime Silk Road in China are globally significant cultural routes, which have contributed a wealth of cultural heritage through their historical development. The study on the cultural heritage of the Grand Canal (East Zhejiang section)-Maritime Silk Road is of great significance for constructing the Grand Canal Cultural Belt and advancing the Belt and Road Initiative. Focusing on the Ningbo area, this study analyzes the spatial and temporal distribution of 1,755 cultural heritage sites over five historical periods and explores the influencing factors through spatial and statistical analysis. The results show that: (1) Ancient buildings, along with modern important historical sites and representative buildings, are the most numerous. The total number of cultural heritages shows an upward trend before the modern period, peaking in the Ming to Qing period. (2) The cultural heritage exhibits an overall aggregated spatial distribution, with varying patterns across different types. The Three-River Estuary is the high-density core area, with the number and density of cultural heritage decreasing as its distance increases. (3) Distribution characteristics of cultural heritage vary across different periods. More recent cultural heritage is increasingly concentrated around the Three-River Estuary. Over time, the center of gravity of cultural heritage has shifted sequentially to the south, southeast, west, and north. (4) The cultural heritage tends to be distributed in plain with low altitude and small slope, and shows strong hydrophilicity. However, it is essentially influenced by human factors.

3. the second half of the research uniqueness section is too cumbersome and unfocused

**Response**: Thank you for your suggestions, we have cut and modified the entire research uniqueness section.

Cultural heritage is a collective term for a multitude of tangible and intangible elements that reflect the past state of human existence, creativity, and the relationship between humans and the environment. These elements, which include monuments and artifacts, collectively form a testament to human civilization. This study analyzes the spatial and temporal distribution characteristics of cultural heritage in Ningbo, a city located at the intersection of the Grand Canal (East Zhejiang section) and the Maritime Silk Road, and explores the factors affecting this distribution.

The Grand Canal and the Maritime Silk Road, two of the most influential cultural routes in human history, have played a key role in the preservation of tangible cultural heritage, representing China’s contribution to global civilization. Previous studies have primarily focused on intangible cultural heritage along the Grand Canal. For instance, Li [4] selected folk songs from the intangible cultural heritage of the Grand Canal and discovered that the spatial distribution of folk songs exhibited a pattern of “distribution along the river with two cores and two belts”; Wang et al. [52] confirmed that traditional skills in the Grand Canal Cultural Belt exhibits a spatial and temporal differentiation from multi-centered dispersal to centralized distribution at the two ends of the canal. However, there has been limited research on the spatial and temporal distribution of tangible cultural heritage, with only a few small data samples. Huang et al. [53] analyzed 104 cultural heritages in six periods along the Suzhou section of the Grand Canal and concluded that the canal’s characteristics significantly shaped the distribution of these heritages. Studies on the cultural heritage along the Maritime Silk Road are even rarer. Niu et al. [54,55] focused on the marine cultural heritage of 11 coastal provinces and cities, exploring the composition of resources, spatial and temporal patterns, and influencing factors of the marine cultural heritage.

Globally, numerous cultural routes like France’s Canal du Midi, Canada’s Canal du Rideau, Oman’s Frankincense Route, Egypt’s Nile River, along with the Ancient Tea Horse Road and the Ancient Shu Road in China have also been studied, though primarily from archaeology [56,57], history [58], and tourism [59,60] perspectives. This study, however, focuses on the material heritage influenced by both the Grand Canal and the Maritime Silk Road, examining how their evolution and interplay have shaped the spatial and temporal distribution of cultural heritage in Ningbo.

The Grand Canal and the Maritime Silk Road, both crucial trade and cultural exchange routes in ancient China and globally, share a close interrelationship. This relationship is not only reflected in the geographical connection, but also in the complementarity and synergy between the two in promoting cultural and economic exchanges, which played an important role in the evolution and development of cultural heritage.

From a geographical standpoint, the Grand Canal and the Maritime Silk Road formed a comprehensive transportation network that connected inland and coastal areas. The Grand Canal facilitated the movement of goods and customs within China, while the Maritime Silk Road enabled international trade. This dual network not only encouraged economic and cultural exchanges but also shaped the diversity of Ningbo’s cultural heritage, evident in its artistic and architectural styles.

From a cultural standpoint, the Grand Canal and the Maritime Silk Road served as conduits for the exchange between Chinese and global civilization. For instance, Indian Buddhism reached Ningbo via the Maritime Silk Road during the late Eastern Han Dynasty, fostering the development of historical Buddhist remains such as Tiantong Temple and Ashoka Temple [61]. Similarly, the spread of Mazu Culture, brought to Ningbo by the Fujian merchants via the Maritime Silk Road, extended throughout Southeast Asia and northern China. The Qing’an Guild Hall (A place for worshipping the sea goddess Mazu) in Ningbo, a symbol of Mazu Culture, reflects the convergence of canal and sea cultures and highlights the far-reaching influence of these routes on Ningbo’s cultural heritage.

4. the selection and categorization of influencing factors lacks some literature support, and it is suggested to add relevant influencing factors literature as a prelude.

**Response**: Thank you for your suggestions, we have added the relevant content at the beginning of the influencing factors analysis. Meanwhile, the references drawn on for the selection and categorization of influence factors are those that have been cited prior to this section, so the articles I have cited in that section have all been cited earlier. In fact, there are many cultural heritage papers that focus on both spatio-temporal distribution and influencing factors. This is sufficient to support this part of my research, so no additional references were added.

The spatial and temporal distribution of the Grand Canal (East Zhejiang section)-Maritime Silk Road cultural heritage shows typical aggregation, with significant differences between different types and periods. Spatial differentiation results from a combination of factors such as nature and human environments. Drawing on existing studies [15,17,20,33,36], six indicators are selected to explore the influence mechanism of cultural heritage distribution through overlay analysis, buffer analysis, and literature analysis.
